# Unveiling the Respiratory Muscle Strength in Duchenne Muscular Dystrophy: The Impact of Nutrition and Thoracic Deformities, Beyond Spirometry

**DOI:** 10.3390/children11080994

**Published:** 2024-08-15

**Authors:** Mine Yuksel Kalyoncu, Yasemin Gokdemir, Cansu Yilmaz Yegit, Muruvvet Yanaz, Aynur Gulieva, Merve Selcuk, Şeyda Karabulut, Neval Metin Çakar, Pinar Ergenekon, Ela Erdem Eralp, Gülten Öztürk, Olcay Unver, Dilsad Turkdogan, Yavuz Sahbat, Ahmet Hamdi Akgülle, Fazilet Karakoç, Bulent Karadag

**Affiliations:** 1 Department of Pediatric Pulmonology, Dr. Lutfi Kirdar City Hospital, Istanbul 34865, Turkey; 2 Department of Pediatric Pulmonology, School of Medicine, Marmara University, Istanbul 34899, Turkey; yasemingokdemir@yahoo.com.tr (Y.G.); g.aynur@ymail.com (A.G.); dr.merveselcuk@gmail.com (M.S.); seydakarabulut@yahoo.com.tr (Ş.K.); nevalmetincakar@hotmail.com (N.M.Ç.); drpergenekon@hotmail.com (P.E.); elaerdem@yahoo.com (E.E.E.); infofaziletkarakoc@gmail.com (F.K.); bkaradag@hotmail.com (B.K.); 3 Department of Pediatric Pulmonology, Çam and Sakura City Hospital, Istanbul 34480, Turkey; cansuuuuyilmaz@gmail.com; 4 Department of Pediatric Pulmonology, Diyarbakir Child Hospital, Diyarbakir 21100, Turkey; muruvvetcenk@gmail.com; 5 Department of Pediatric Neurology, School of Medicine, Marmara University, Istanbul 34899, Turkey; gulten@thomas.md (G.Ö.); olcaymd@hotmail.com (O.U.); dturkdogan@hotmail.com (D.T.); 6 Department of Orthopaedic Surgery and Traumatology, School of Medicine, Marmara University, Istanbul 34899, Turkey; yavuzsahbat@gmail.com (Y.S.); ahmethamdiakglle@yahoo.com (A.H.A.)

**Keywords:** Duchenne muscular dystrophy, sniff nasal inspiratory pressure, supine spirometry, diaphragm

## Abstract

Background/Objectives: Duchenne muscular dystrophy (DMD) is the most prevalent progressive muscular dystrophy, and the guidelines recommend the regular assessment of respiratory muscle function. This study aimed to assess the relationship between maximum inspiratory pressure (MIP), maximum expiratory pressure (MEP) and sniff nasal inspiratory pressure (SNIP) measurements and upright-supine spirometry parameters in children with DMD, the predictability of upright–supine spirometry in terms of diaphragm involvement, and the impact of nutrition on muscle strength. Methods: This prospective cross-sectional study examined patients with DMD by comparing upright and supine FVC, MIP, MEP, and SNIP measurements. The effects of the ambulatory status, kyphoscoliosis, chest deformity, and low BMI on respiratory parameters were investigated. Results: Forty-four patients were included in the study. The mean patient age was 10.8 ± 2.9 years. Twenty-five patients were ambulatory. A significant decrease in FVC, FEV1, and FEF25–75 values was detected in the supine position in both ambulatory and non-ambulatory patients (*p* < 0.05). All patients had low MIP, MEP, and SNIP measurements (less than 60 cm H_2_O). MIP, MEP, and SNIP values were significantly lower in patients with a low BMI than in those without (*p* < 0.05). Conclusions: To accurately assess respiratory muscle strength, supine FVC should be combined with upright FVC, MIP, MEP, and SNIP measurements. It is crucial to regularly screen patients for nutrition, as this can significantly affect respiratory muscle function during pulmonology follow-up.

## 1. Introduction

Duchenne muscular dystrophy (DMD) is the most common progressive muscular dystrophy, presented in early childhood with lower extremity muscle weakness [1]. Respiratory muscles are affected over time, resulting in decreased lung compliance, an ineffective cough, and recurrent infections [1]. Respiratory complications are a significant cause of morbidity and mortality in patients with DMD, and respiratory care is essential [2].

The American Thoracic Society (ATS) recommends the routine monitoring of respiratory muscle functions to guide respiratory management [3]. Forced vital capacity (FVC) is commonly used to measure the strength of inspiratory and expiratory muscles in DMD but is highly dependent on the patient’s motivation and understanding. It can be challenging for patients to perform appropriate and accurate testing because the average intelligence quotient is one standard deviation below the average [4]. Maximum inspiratory pressure (MIP), maximum expiratory pressure (MEP), sniff nasal inspiratory pressure (SNIP), and peak cough flow (PCF) are useful tools for assessing the respiratory muscle strength in patients with DMD [5,6].

Compared to transdiaphragmatic pressure measurement, MIP, MEP, and SNIP are non-invasive and relatively simple to perform [7]. SNIP is administered by “sniffing”, a natural maneuver that does not require a mouthpiece and can be administered to children as young as four [7]. Some cutoff values for MIP and MEP are commonly used in the respiratory management of patients with DMD [2]. For adults and adolescents with DMD, ATS recommends cough support when MEP is <60 cm H_2_O and non-invasive ventilation (NIV) support when MIP is <60 cm H_2_O [2]. However, studies on MIP and MEP in children with DMD are limited. There are no specific cut-off values for children in the guidelines [8,9,10]. Studies on SNIP have generally been conducted in healthy individuals, with very few studies on children with DMD [9,10,11,12,13].

The inspiratory muscles, especially the diaphragm, progressively deteriorate as DMD progresses. Therefore, specific diaphragmatic outcome measures are required to assess the disease’s progression [14]. There are several ways to evaluate the involvement of the diaphragm, one of which is to compute the difference between FVC in the upright and supine positions [15]. Few studies have assessed the relationship between upright and supine FVC values and respiratory muscle strength [15,16,17,18].

The purpose of this study was to evaluate the relationship between MIP, MEP, and SNIP measurements and upright–supine spirometry parameters in children with DMD, the predictability of upright–supine spirometry in terms of diaphragm involvement, and the effect of nutrition on muscle strength.

## 2. Materials and Methods

### 2.1. Patients and Study Protocol

This prospective, cross-sectional study was conducted between January and December 2022. Patients who had been regularly examined at the pediatric pulmonology outpatient clinic for the last one year, were diagnosed with DMD through genetic testing, were able to perform spirometry, and gave consent to participate in the study were included in this study. The study excluded children with respiratory conditions such as asthma, obstructive sleep apnea, nasal polyposis, or any other condition that could influence the spirometry results and necessitate NIV, or children with autism and cognitive dysfunction who were not sufficiently mentally competent to follow instructions.

If the patient had a common cold, evaluations were performed three weeks after recovery. The physical examination was performed by the same pediatric pulmonologist. Patients’ medical history was obtained from the medical records. Patients who could walk unassisted for 10 m without a cane or brace were considered ambulatory. Loss of ambulation was defined as continuous wheelchair use [19].

### 2.2. Measurements

The patients’ weight and height were measured, and the body mass index (BMI), expressed as kg/m^2^, was computed by dividing the weight by the square of the height. For non-ambulatory patients, arm spans were measured [20]. The BMI measurements of our patients were made by considering the measurements of Turkish children [21]. The BMI *z*-score represents the number of standard deviations from the average weight of the reference group and the individual’s BMI. The 2000 Center for Disease Control and Prevention Growth Charts were used to calculate the BMI *z*-scores for boys aged 2–20 years, which were then converted into BMI percentile (BMIp) units for easier interpretation and the patients were classified into two groups based on their BMIp: the low BMI group (less than the 50th percentile) and the normal BMI group (above the 50th percentile) [22]. Pulmonary function tests were performed by qualified respiratory therapists using spirometry (Winspiro PRO 2.8 MIR, Rome, Italy) following the ATS/European Respiratory Society (ERS) standards [23]. FVC, FEV1, FEV1/FVC, and FEF25–75 were measured and are presented as percentages of the predicted values [24].

MIP, MEP, and SNIP measurements were performed following the ATS/ERS standards [23]. Oxygen saturation was recorded using a pulse oximeter (Konica Minolta Pulsox-300i; Stowood Scientific Instruments, Oxford, UK). Following spirometry, MIP, MEP, and SNIP tests (MicroRPM device, Vyaire, Mettawa, IL, USA) were performed after an hour of rest. An MIP, MEP, and SNIP of >60 cm H_2_O were considered normal for pediatric patients with DMD [2,8,10,11].

The postural FVC difference (ΔFVC) was used as a parameter of diagram involvement, which was calculated by subtracting the supine FVC from the upright FVC. The percentage of ΔFVC = (FVC upright − FVC supine)/FVC upright × 100 [15]. Those with a ΔFVC difference of more than 7.5% were grouped as those with diaphragm involvement and those with less than 7.5% were categorized as without diaphragm involvement [15].

### 2.3. Orthopedic Assessment

Each patient underwent comprehensive assessments conducted by a single pediatric orthopedic surgeon. Because they could affect spirometry, an orthopedic surgeon meticulously examined various clinical factors, such as scoliosis, kyphosis, and chest deformities (e.g., excavatus and carinatus) during outpatient clinic visits. Thoracolumbar scoliosis was diagnosed based on a Cobb’s angle exceeding 10° and a T2–T12 sagittal (kyphosis) angle, as observed on an upright anterior–posterior (AP) view of the chest X-ray [25]. The sagittal angle of the T2–T12 vertebrae was measured using the sagittal view. Individuals exhibiting angles exceeding 45° were categorized as presenting kyphosis [26].

### 2.4. Ethical Approval

Informed consent was obtained from the patient’s caregivers. Approval for this study was obtained from the Institutional Ethical Committee of Marmara University School of Medicine (09.2022.1140).

### 2.5. Statistical Analysis

All test variables were first determined for normality. If the distribution was normal, an unpaired parametric t-test was performed to assess the differences between the two groups. An equivalent nonparametric Mann–Whitney U test was performed for variables without normal distribution. Pearson correlation was performed for normally distributed variables, such as %FVC, %FEV1, %FEF2575, MIP, and SNIP. Otherwise, the Spearman correlation was performed, namely for MEP. All statistical analyses were performed using SPSS 10.0 (SPSS, Chicago, IL, USA) software. A *p*-value < 0.05 was considered significant.

## 3. Results

### 3.1. Study Population

Of the 51 patients, seven were excluded from the study because they could not cooperate with the pulmonary function test. Forty-four patients were included. The mean age of the patients was 10.8 ± 2.9 years, and 42 (95.5%) were male. Of these, 25 were ambulatory, and 19 were non-ambulatory. There were significant differences between these patients in terms of age, COBB angle, the number of people with scoliosis, the number of people with kyphosis, orthopedic and cardiological problems, and the duration of steroid use. The median age at diagnosis in all patients was 3.5 years (25–75 percentile, 1.6–5 years), and there was no difference between the ambulatory and non-ambulatory groups (*p* > 0.05). All patients were treated with methylprednisolone therapy with a median treatment duration of 4 years. (25–75 percentile, 2–6 years). Thirty-eight patients were still receiving steroid treatment, and six patients had discontinued the use of steroids in the last year due to side effects. Table 1 summarizes the patients’ characteristics and other relevant features.

### 3.2. Respiratory Assessments

Twenty-nine patients had normal FVC measurements (≥80%), but all of them had low MIP, MEP, and SNIP results (less than 60 cm H_2_O). The upright and supine spirometry values of the ambulatory and non-ambulatory patients are presented in Table 2. A significant decrease in the FVC, FEV1, and FEF25–75 values was detected in the supine position in ambulatory and non-ambulatory patients (*p* < 0.05) (Table 2). The ΔFVC had a median of 3.7% (25–75p, 1.1–9.7) in ambulatory patients and a median of 5.7% (25–75p, 1.0–8.5) in non-ambulatory patients; there was no significant difference between the two groups (*p* > 0.05).

All patients demonstrated reductions in MIP, MEP, and SNIP (less than 60 cm H_2_O), regardless of diaphragm involvement. However, their spirometry results for FVC, FEV1, and FEF2575 were found to be normal. There was a significant difference in the SNIP measurements between the two groups with and without diaphragmatic involvement (*p* < 0.05) (Table 3).

**Figure 1:**
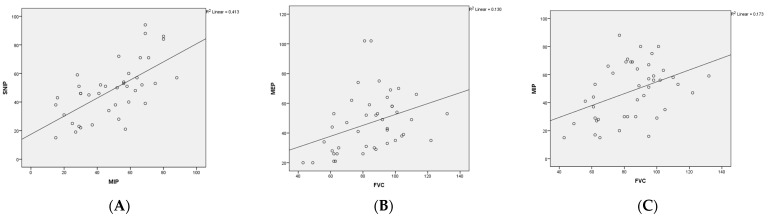
Correlation between MIP, MEP, SNIP and FVC in children with DMD. (**A**) Relationship between MIP (cm H_2_O) and SNIP (cm H_2_O) at baseline in the children with DMD. Pearson test r: 0.64 (*p* = 0.000); (**B**) Relationship between MEP (cm H_2_O) and FVC (%) at baseline in the children with DMD. Spearman test r: 0.44 (*p* = 0.003); (**C**) Correlation between MIP (cm H_2_O) and FVC (%) at baseline in the children with DMD. Pearson test r: 0.41 (*p* = 0.006). MIP (cm H_2_O), maximum inspiratory pressure; MEP (cm H_2_O), maximum expiratory pressure; FVC (%), forced vital capacity; DMD, Duchenne muscular dystrophy.

There was a significant and positive correlation between MIP and FVC (Pearson r = 0.41, *p* = 0.006) and MEP and FVC (Spearman r = 0.44, *p* = 0.003) in children with DMD. Also, there was a significant and positive correlation between MIP and SNIP (Pearson r = 0.64, *p* = 0.000), but there was no significant correlation between FVC and SNIP (Pearson r = 0.30, *p* = 0.055) (Figure 1).

### 3.3. Effect of Kyphoscoliosis, Chest Deformity and BMIp Status

No significant change was detected in MIP and MEP measurements in patients with and without kyphoscoliosis (*p* > 0.05); only SNIP was significantly lower in patients with kyphoscoliosis (*p* < 0.05). The MIP and SNIP measurements were significantly lower in patients with chest deformities than those without (*p* < 0.05).

Table 4 presents the respiratory parameters based on BMI status and kyphoscoliosis. The MIP, MEP, and SNIP values were lower than normal (<60 cm H_2_O) in both the low and normal BMI groups. Additionally, the MIP, MEP, and SNIP values were significantly lower in the low BMI group than in the normal BMI group (*p* < 0.05).

Sixteen patients had both kyphoscoliosis and a low BMI, and their MIP, MEP, and SNIP values were lower than those in the group without both conditions. The median COBB angle in the kyphoscoliosis and low BMI groups was 11° (25–75p, 6–16°), which was significantly higher than that in the non-kyphoscoliosis and low BMI groups (*p* < 0.05) (Table 4).

## 4. Discussion

This study revealed that the MIP, MEP, and SNIP values were low in DMD patients with normal FVC values. We also found a significant decrease in FVC, FEV1, and FEF2575 values in the supine position in both ambulatory and non-ambulatory patients. In addition, a low BMI was associated with low MIP, MEP, and SNIP values. This is the first study to assess the association between respiratory muscle strength and the upright and supine FVC.

Several studies in adult patients with motor neuron disease have reported that the MIP, SNIP, and MEP may be more sensitive than spirometry in detecting early respiratory muscle dysfunction [27,28,29], and guidelines recommend using these measurements during routine follow-up [8]. However, only a few studies have evaluated and compared MIP, MEP, SNIP, and upright and supine spirometry in children with DMD [15,16,17,18]. In the current study, the MIP, MEP, and SNIP were low (less than 60 cm H_2_O) in ambulatory and non-ambulatory patients. Although it was lower in non-ambulatory patients, the decrease was not significant. Levine et al. found significant differences in the MIP and MEP measurements between the groups based on ambulation status [30]. In another study conducted with 53 patients, significant differences in the MIP and MEP values were observed between groups [31]. Both studies, including those involving children and adults, had large sample sizes. Therefore, our results may be related to the small sample size of the present study.

Although diaphragmatic function is relatively well preserved in patients with DMD, it has been reported that the diaphragm is affected in advanced disease [32,33]. Invasive and non-invasive tests are available to measure diaphragm strength. Upright and supine FVC are commonly used in clinical practice [34,35]. Our results demonstrated a significant reduction in FVC, FEV1, and FEF2575 in the supine position in ambulatory and non-ambulatory patients. However, this decrease (ΔFVC) was not more than 20%, which is defined as diaphragm involvement [34]. These results are consistent with those of previous studies [16,32,36]. As has been observed in other neuromuscular diseases, the supine position is thought to accentuate actual respiratory defects [34]. An increasing ΔFVC may be an early sign of poor respiratory outcomes, and these patients should be closely monitored [37].

Few studies have investigated the relationship between nutritional status and respiratory outcomes in patients [38,39,40,41]. Fayssoil et al. reported that the MIP and MEP were positively correlated with BMI in adult patients with DMD receiving NIV support [38]. Chew et al. showed that the FVC and FEV1 were positively associated with BMI in children with DMD [41] Our study found that low MIP, MEP, and SNIP values were related to a low BMI, although the spirometry values were within the normal range. These results emphasize that nutritional status is closely associated with respiratory muscle strength, that multidisciplinary collaboration, including gastroenterologists and dietitians, is crucial for managing these patients, and that steroid treatment for DMD slows the progression of muscle weakness.

Patients with scoliosis and chest deformities are at a higher risk of developing restrictive lung disease and experiencing a decline in respiratory muscle strength [42]. However, further research is needed to determine the most effective method of evaluating the impact of these conditions on respiratory function. Our study found that patients with kyphoscoliosis had a significantly lower normal FVC compared to those without kyphoscoliosis. In a previous study, the MIP and MEP measurements were not affected by scoliosis [42]. On the other hand, SNIP measurements might help differentiate neuromuscular scoliosis from idiopathic scoliosis [43]. However, more research is needed to determine the specific factors affecting SNIP measurements, as other features may be present in the same patient group. Regression analysis was not possible due to the small number of patients.

The main limitation of this study was the small sample size of subgroups such as those with kyphoscoliosis or a low BMI, although the total number of pediatric patients was sufficient. Despite this, we were able to obtain informative data. Another limitation was the variability in steroid doses and durations among our patients, which prevented us from assessing the impact of steroid treatment on respiratory outcomes. However, our study was the first to compare upright supine FVC with MIP, MEP, and SNIP measurements in children with DMD. Although upright–supine spirometry has been recommended for screening diaphragmatic weakness in children with neuromuscular diseases, most studies on this topic have been conducted in adults [16,17,33,44]. Therefore, the findings of our study are significant, and it provides crucial information for future research on DMD.

## 5. Conclusions

This study showed that respiratory muscle strength in patients with DMD should be assessed using supine and upright FVC, MIP, MEP, and SNIP. The addition of supine FVC to routine respiratory evaluation is essential because of the predictive value of the diaphragm. A low BMI affects respiratory muscle function; therefore, it is important to inform pediatric pulmonologists, gastroenterologists, and dietitians of the patient’s assessment. 

## Figures and Tables

**Table 1:** Demographics and clinical characteristics of the patients (n:44).

	All Patients (n:44)	Ambulatory Patients (n:25)	Non-Ambulatory Patients (n:19)	*p* Value
Age (years), (mean ± std)	10.8 ± 2.9	9.2 ± 2.1	13 ± 2.3	0.000 *
Male, n (%)	42 (95.5)	23 (92)	19 (100)	0.212
Body-mass index percentile (BMIp) (median) (25–75 percentile)	52.5 (12.9–86.4)	49.6 (27–81.5)	55.5 (0.9–84)	0.499
Age at diagnosis (years), (median) (25–75 percentile)	3.5 (1.6–5)	4 (1.8–5)	3 (1.5–5)	0.426
Consanguineous marriage, n (%)	11 (25)	5 (20)	6 (31.6)	0.385
Family history of DMD patients, n (%)	11 (25)	8 (32)	3 (15.8)	0.224
Kyphosis, n (%)	13 (29.5)	11 (44)	2 (10.5)	0.017 *
Scoliosis, n (%)	12 (27.3)	2 (8)	10 (52.6)	0.001 *
COBB angle, °, (median) (25–75 percentile)	6 (2–11)	5 (0–7.7)	10 (4–16)	0.005 *
Chest deformity, n (%)	12 (27.3)	4 (16)	8 (42.1)	0.057
Other orthopedic problems (limb contractures, pes equinovarus etc…) n (%)	25 (56.8)	9 (36)	16 (84.2)	0.002 *
Cardiological problems, n (%)	6 (13.6)	0 (0)	6 (31.6)	0.003 *
Venous blood gas pCO_2_ (mmHg) (mean ± std)	37.7 ± 4.1	36.8 ± 3.1	39.0 ± 5.1	0.450
Steroid treatment, (years), (median) (25–75 percentile)	4 (2–6)	3 (1.5–5)	6 (4–11.1)	0.005 *

*: *p* < 0.05 significancy.

**Table 2:** Upright–supine pulmonary function tests evaluation according to the ambulation status (n:44).

	Ambulatory Patients (n:25)			Non-Ambulatory Patients (n:19)		
	Upright Position	Supine Position	*p* Value	Upright Position	Supine Position	*p* Value
FVC (%) (mean ± std)	92.3 ± 17.4	87.3 ± 19.2	0.000 *	75.7 ± 18.9	71.1 ± 19.0	0.021 *
FEV1 (%) (mean ± std)	98.2 ± 17.0	91.8 ± 18.9	0.000 *	83.8 ± 18.5	77.6 ± 18.8	0.002 *
FEV1/FVC (%) (mean ± std)	103.3 ± 4.2	102.4 ± 4.8	0.330	107.0 ± 6.1	106.4 ± 6.0	0.457
FEF2575 (%) (mean ± std)	100.8 ± 14.3	92.8 ± 16.3	0.001 *	97.1 ± 25.9	88.3 ± 26.1	0.040 *
MIP (cm H_2_O) (mean ± std)	51.0 ± 20.2	NA	NA	44.5 ± 19.3	NA	NA
SNIP (cm H_2_O) (mean ± std)	52.6 ± 15.7	NA	NA	42.1 ± 23.3	NA	NA
MEP (cm H_2_O) (median, 25–75p)	49 (35–67.5)	NA	NA	39 (26–53.2)	NA	NA

*: *p* < 0.05 significancy; NA: not available.

**Table 3:** Assessment with SNIP, MIP, and MEP for patients according to diaphragmatic involvement.

	Postural FVC Difference < 7.5% (n:27)	Postural FVC Difference > 7.5% (n:17)	*p* Value
MIP (cm H_2_O) (mean ± std)	52.38 ± 19.79	43.81 ± 18.13	0.168
SNIP (cm H_2_O) (mean ± std)	53.40 ± 19	41.93 ± 17.54	0.049 *
MEP (cm H_2_O) (median, 25–75p)	47 (31–59)	38 (28.50–53.75)	0.407
FVC (%) (mean ± std)	85.07 ± 17.51	85.35 ± 23.50	0.838
FEV1 (%) (mean ± std)	94 ± 15.73	91.53 ± 21.39	0.665
FEV1/FVC (%) (mean ± std)	104.96 ± 5.71	104.88 ± 5.04	0.946
FEF2575 (%) (mean ± std)	98.80 ± 19.48	103.17 ± 16.35	0.449

*: *p* < 0.05 significancy.

**Table 4:** Respiratory parameters according to BMIp status and kyphoscoliosis–low BMIp association (n:44).

BMIp Status				Kyphoscoliosis and Low BMIp Association		
	Low BMI Group (n:28)	Normal BMI Group (n:16)	*p* value	Group with Kyphoscoliosis and Low BMI (n:16)	Group without Both Kyphoscoliosis and Low BMI (n:30)	*p* Value
MIP (mean ± std)	39.6 ± 18.6	61.7 ± 14.0	0.000 *	37.1 ± 16.4	53.7 ± 19.3	0.010 *
MEP (median, 25–75p)	35 (26–49.7)	54 (47–64)	0.001 *	36.5 (26–44.5)	52 (33–65)	0.010 *
SNIP (mean ± std)	40.9 ± 14.3	61.7 ± 21.7	0.001 *	38.5 ± 12.8	53.7 ± 20.7	0.018 *
FVC (%) (mean ± std)	84.1 ± 22.4	85.1 ± 14.9	0.854	85.3 ± 27.3	85.1 ± 15.6	0.960
FEV1 (%) (mean ± std)	91.0 ± 21.5	92.0 ± 14.0	0.867	92.5 ± 26.7	91.7 ± 14.5	0.801
FEV1/FVC (%) (mean ± std)	105.4 ± 4.7	104.5 ± 6.6	0.627	105.1 ± 4.1	104.8 ± 5.9	0.859
FEF2575 (%) (mean ± std)	95.2 ± 17.1	104.1 ± 22.8	0.159	95.2 ± 20.3	101.1 ± 19.9	0.743
COBB angle, °, (median) (25–75p)	5.5 (1.2–8)	9 (4–12)	0.107	11 (6–16)	3 (1–6.5)	0.000 *

* *p* < 0.05 significancy.

## Data Availability

The data presented in this study are available on request from the corresponding author due to privacy.
